# Monitoring cognitive functioning in multiple sclerosis will trigger anxiety in patients: Commentary

**DOI:** 10.1177/13524585241261200

**Published:** 2024-06-16

**Authors:** Sarah A Morrow, Laura Hancock

**Affiliations:** Hotchkiss Brain Institute, University of Calgary, Calgary, AB, Canada; Neurological Institute, Cleveland Clinic, Cleveland, OH, USA

In this controversies, we address whether regular cognitive screening triggers anxiety in people with multiple sclerosis (PwMS). It is now considered fact that cognitive impairment (CI) is a frequent consequence of MS, can have significant negative consequences, and that regular cognitive screening leads to early identification and intervention.^1,2^ Yet, the specifics of cognitive screening are still up for discussion.

Although there are concerns that cognitive screening could cause anxiety, we are not aware of literature examining this phenomenon in PwMS. Krupp and O’Neill note the potential for anxiety given that screening could lead to false-positive outcomes, due to a variety of factors, including administration errors. However, ignoring cognitive symptoms in clinic because of imperfect screening methods is unacceptable. Krupp and O’Neill state that the Symbol Digit Modalities Test (SDMT), a validated and recommended tool for cognitive screening in MS, is flawed because it is sensitive to factors besides slowed processing speed, which is indeed true. However, there is a value in using a screening tool as a means to measure change over time. There is a wealth of data regarding how PwMS perform on the SDMT longitudinally^3^ and what constitutes clinically meaningful change over time,^4–6^ thereby assisting the clinician in knowing when more detailed assessment may be needed.

Ignorance is not always bliss; anxiety could develop in response to job loss or other consequences of CI that are missed without screening. PwMS highlight that “invisible” symptoms, such as CI, are one of the greatest unmet needs, and open discussions regarding these symptoms are imperative but not always offered.^7^ As noted by Klein, open communication and engaging in problem-solving can lead to improved self-efficacy, helping empower PwMS to take an active role in monitoring/discussing cognition. Taking insights from the cancer literature, Klein notes that anticipatory anxiety (negative feelings/worry experienced when facing uncertainty) stems from the heightened anxiety regarding a specific diagnosis. If PwMS can feel empowered to engage in a discussion regarding these concerns, this anticipatory anxiety may be negated.

Krupp and O’Neill’s concern regarding the misinterpretation of cognitive tests is valid; yet there needs to be a clear distinction between screening and diagnostic tests; these are not the same. Screening via the use of a properly administered, reliable, and valid tool can lead to early identification of impairment or change over time and timely intervention.

The clinical care of PwMS should be focused on disease management, alleviating symptoms and maintaining functional ability. If an impairment is found on a screening test, whether due to CI or possible another interfering cause, we need to address these symptoms to improve the function and quality of life (QoL) of PwMS. For example, if a PwMS had difficulty with gait, and we were unsure if this was due to weakness or spasticity, would we simply ignore it because the underlying cause was unclear? The answer is a resounding no. However, as noted by Krupp and O’Neill, screening should not comprise just one single test without context for interpretation, such as talking to family members. Using a screening test as a stepping stone to explore what is being experienced and identify the underlying cause is imperative, important to the overall QoL of PwMS.
